# Impact of a high loading dose of amikacin in patients with severe sepsis or septic shock

**DOI:** 10.1186/s13613-016-0211-z

**Published:** 2016-11-02

**Authors:** Nicolas Allou, Astrid Bouteau, Jérôme Allyn, Aurélie Snauwaert, Dorothée Valance, Julien Jabot, Bruno Bouchet, Richard Galliot, Laure Corradi, Philippe Montravers, Pascal Augustin

**Affiliations:** 1 Réanimation polyvalente, Hôpital Felix Guyon, Centre Hospitalier Universitaire Felix Guyon, Allée des Topazes, Bellepierre, 97405 Saint Denis, France; 2 Département d’Anesthésie Réanimation, AP-HP, Centre Hospitalier Universitaire Bichat-Claude-Bernard, 46, rue Henri-Huchard, 75018 Paris, France

**Keywords:** Amikacin, Pharmacokinetics/pharmacodynamics, Severe sepsis/septic shock

## Abstract

**Background:**

The therapeutic effect of aminoglycosides is highest and optimal when the peak plasma concentration (*C*_max_)/minimal inhibitory concentration (MIC) ratio is between 8 and 10. The French guidelines recommend to use high doses of aminoglycosides for empiric antibiotic therapy in patients suffering from severe sepsis or septic shock. In clinical practice, the recommended target is an amikacin *C*_max_ between 60 and 80 mg/L, which corresponds to approximately 8 times the MIC breakpoint, as defined by the European Committee on Antimicrobial Susceptibility Testing. The aim of this study was to assess the incidence and impact on mortality of an amikacin concentration between 60 and 80 mg/L in patients suffering from severe sepsis or septic shock.

**Methods:**

This was a prospective observational cohort study conducted in two intensive care units (ICU). Patients receiving amikacin at a loading dose of 30 mg/kg for severe sepsis or septic shock were enrolled in the cohort. The target *C*_max_ for amikacin was between 60 and 80 mg/L, as recommended by French guidelines (i.e. *C*_max_/MIC breakpoint = 8–10).

**Results:**

Over the study period, the amikacin *C*_max_ was <60 mg/L, between 60 and 80 mg/L, and >80 mg/L in 20 (18.2%), 46 (41.8%) and 44 (40%) of the 110 selected patients, respectively. Mortality rate was 40, 28.3 and 56.8% in the groups of patients with *C*_max_ < 60 mg/L, 60 mg/L < *C*_max_ < 80 mg/L and *C*_max_ > 80 mg/L, respectively. Following multivariate analysis, mortality rate was significantly lower in the group of patients with amikacin *C*_max_ between 60 and 80 mg/L than in the group of patients with amikacin *C*_max_ > 80 mg/L (*P* = 0.004). The multivariate analysis also revealed that the factors independently associated with a higher in-ICU mortality rate were age (*P* = 0.02) and norepinephrine dose (*P* = 0.0001).

**Conclusions:**

With a loading dose of 30 mg/kg of amikacin, concentration was potentially suboptimal (*C*_max_ < 60 mg/L) in only 18.2% of patients. The pharmacodynamic target (60 mg/L < *C*_max_ < 80 mg/L) recommended by French guidelines was reached in 41.8% of patients and was associated with reduced in-ICU mortality. But amikacin overexposure (i.e. *C*_max_ > 80 mg/L) was frequent and potentially associated with increased mortality.

## Background

The usefulness of antimicrobial combination therapy, such as the use of aminoglycosides in patients with severe sepsis or septic shock, remains controversial [1, 2]. Nevertheless, early and appropriate antimicrobial therapy in septic shock patients has been reported to reduce in-hospital mortality [2]. In view of this, aminoglycosides are often combined with another antibiotic in patients with severe sepsis or septic shock in order to broaden the treatment’s spectrum of activity and obtain bactericidal synergy. The therapeutic effect of aminoglycosides is highest and optimal when the peak plasma concentration (*C*_max_)/minimal inhibitory concentration (MIC) ratio is between 8 and 10 [3]. However, in patients with severe sepsis or septic shock, aminoglycosides are frequently used in empiric antimicrobial treatment without the causal micro-organisms and their MICs being identified. The French guidelines recommend to use high doses of aminoglycosides for empiric antibiotic therapy in patients suffering from severe sepsis or septic shock, and, specifically, doses of 25–30 mg/kg in the case of amikacin [4, 5]. In clinical practice, the recommended target is an amikacin *C*_max_ between 60 and 80 mg/L [4], which corresponds to approximately 8–10 times the MIC breakpoint, as defined by the European Committee on Antimicrobial Susceptibility Testing (EUCAST) of *Pseudomonas aeruginosa* and Enterobacteriaceae [5]. Previous studies have shown that a loading dose of 25 mg/kg frequently leads to a suboptimal amikacin *C*_max_ (>30% of patients with *C*_max_ < 60 mg/L) [6, 7], prompting an increase in the loading dose to 30 mg/kg without improving outcomes [8, 9]. Yet the dose of 30 mg/kg has been evaluated in a limited number of patients (*n* < 50) [8, 9]. The aim of this study was to assess the incidence and impact on mortality of an amikacin concentration between 60 and 80 mg/L in patients suffering from severe sepsis or septic shock.

## Methods

The present observational study was approved by the Ethics Committee of Félix Guyon University Hospital (R15003). The requirement to obtain written informed consent from patients was waived, as the study was non-interventional and followed our usual protocol. However, all patients or their legally authorised representative was verbally informed about the process of data collection and could refuse to participate.

The reporting of this study complies with the Strengthening the Reporting of Observational studies in Epidemiology recommendations statement [10].

### Selection of the study sample

This prospective observational cohort study was conducted from April 2015 to December 2015 in one mixed medical/surgical ICU and in one surgical ICU at two French university hospitals.

All patients were over 18 years old and treated for severe sepsis or septic shock with a combination therapy of amikacin at a loading dose of 30 mg/kg (±2.5 mg/kg) on the day of enrolment.

The first dose of amikacin was calculated using the total body weight of the day for patients with a body mass index <30 kg/m^2^. For patients with a body mass index ≥30 kg/m^2^, the dose was calculated using the adjusted body weight, which was calculated as follows: ideal body weight (height (cm) − 100 − [height (cm) − 150]/4 for male gender and height (cm) − 100 − [height (cm) − 150]/2 for female gender) + 0.4 [total body weight at admission-ideal body weight].

Severe sepsis and septic shock were defined following the Surviving Sepsis Campaign guidelines [11].

Only the first day of amikacin administration was studied.

The prescribed duration of the continuous infusion was 30 min. Measurements of *C*_max_ and trough concentration were taken 30 min and 24 h after the end of infusion. All samples were analysed immediately. Amikacin dosages were measured using a fluorescence polarisation immunoassay [12].

Trough concentration was considered elevated if >2.5 mg/L and was assessed only if treatment length was >5 days or in cases of renal insufficiency, as recommended by French guidelines [4, 6].

The exclusion criteria were: treatment with amikacin within 7 days prior to inclusion; inappropriate dose of amikacin (<27.5 or >32.5 mg/kg); inappropriate duration of amikacin infusion (<25 or >35 min); and inappropriate delays in measuring the amikacin *C*_max_ (<20 or >40 min after the end of infusion).

### Data collected and study endpoint

Patient comorbidities at ICU admission were recorded on inclusion.

After administering the loading dose of 30 mg/kg of amikacin, the main endpoint was to evaluate the impact of the amikacin *C*_max_ on the in-ICU mortality of patients with severe sepsis or septic shock.

### Statistical analysis

Results were expressed as total number (percentage) for categorical variables and as median [25th–75th percentiles] for continuous variables. Continuous variables were compared using the Mann–Whitney test or the Kruskal–Wallis test, as appropriate. Categorical variables were compared using the Chi-square test or the Fisher’s exact test, as appropriate. Risk factors for in-ICU mortality in bivariate analysis with *P* < 0.1 were entered into a multivariate logistic regression analysis using backward selection with *P* < 0.05.

We considered that 50 patients were needed in the non-survivors group to perform a multivariate analysis with ten variables [13]. Based on an in-ICU mortality rate of 50% in patients with severe sepsis or septic shock [6], we calculated that 100 patients had to be included in the study cohort for there to be a minimum of 50 patients in the non-survivors group. Collinearity between independent factors was investigated. When identified, the most clinically relevant factor was chosen for use in the multivariate model. A *P* value <0.05 was considered significant. Analyses were performed using SAS statistical software (8.2, Cary, NC, USA).

## Results

### Study population

Over the study period, 921 patients were hospitalised, of which 150 (16.3%) received amikacin for severe sepsis or septic shock. Among the latter, 40 were excluded: 15 had received aminoglycosides in the previous seven days; 15 had received an inappropriate dose of amikacin; and 10 had an incorrect *C*_max_ measurement taken. The remaining 110 patients formed the cohort.

Patient characteristics on ICU admission and study inclusion are shown in Tables 1, 2 and 3.

**Table 1:** Baseline patient characteristics at intensive care unit admission and at inclusion

Characteristics	Total (*n* = 110)	Amikacin *C*_max_ (mg/L)			*P*
		<60 (*n* = 20)	60–80 (*n* = 46)	>80 (*n* = 44)	
At ICU admission					
Age (years old)	61 [51–70]	57. 5 [50.5–69]	63 [45–69]	62 [55.5–70]	0.521
Male sex	78 (70.9)	14 (70)	33 (71.7)	31 (70.5)	0.986
Weight (kg)	70 [58.2–80]	60 [52.5–73.5]	70 [58.2–80]	72.5 [65–82]	0.029
Body mass index (kg/m^2^)	24.6 [20.7–28]	21.1 [18.5–25.7]	23.7 [20.8–27.3]	26.6 [21.9–29]	0.023
APACHE II	23 [18–28]	25 [21–28]	23 [17–28]	22 [18–26]	0.309
Simplified Acute Physiology Score 2	54 [41–68]	62 [47–70]	54 [44–63]	52 [40–67]	0.361
History of congestive heart failure	12 (10.9)	1 (5)	8 (17.4)	3 (6.9)	0.177
Liver cirrhosis	4 (3.6)	0	2 (4.3)	2 (4.5)	0.63
Chronic obstructive pulmonary disease	26 (23.6)	5 (25)	10 (21.7)	11 (25)	0.924
Chronic kidney disease requiring dialysis	9 (8.2)	1 (5)	2 (4.3)	6 (13.6)	0.233
Cancer (<3 months)	16 (14.5)	4 (20)	10 (21.7)	2 (4.5)	0.051
Diabetes mellitus	39 (35.5)	7 (35)	12 (26.1)	20 (45.5)	0.158
Immunodepression	24 (21.8)	6 (30)	9 (19.6)	9 (20.5)	0.616
At the day of aminoglycoside administration					
Day since admission	1 [0–5]	1 [0–6]	0.5 [0–5]	1.5 [0–6.5]	0.834
Emergent surgery	25 (22.7)	9 (45)	12 (26.1)	4 (9.1)	0.005
Sequential Organ Failure Assessment score	10 [8–11]	10 [9–11]	10 [7–12]	10 [8–12]	0.950
Weight (kg)	70 [60–80]	66 [55.5–79]	71 [58.2–80]	72 [65.5–82]	0.104
Weight gain since ICU admission (kg)	0 [0–3]	0 [0–3]	0 [0–2]	0 [0–2]	0.847
Extracorporeal membrane oxygenation	15 (13.6)	4 (20)	5 (10.9)	6 (13.6)	0.611
Catecholamines	89 (80.9)	16 (80)	36 (78.3)	37 (84.1)	0.776
Norepinephrine (µg/kg/min)	0.44 [0.1–0.93]	0.88 [0.11–1.64]	0.46 [0.09–0.83]	0.41 [0.11–0.81]	0.343
PaO_2_/FiO_2_ ratio	177 [122–264]	155 [90–230]	203 [140–289]	159 [118–258]	0.234
Renal replacement therapy	40 (36.4)	12 (60)	15 (32.6)	13 (29.5)	0.05
Glomerular filtration rate (mL/min)	45 [18.6–86.2]	22.1 [0–90.4]	43.6 [24.6–86]	49.7 [22.5–83.9]	0.583
Lactate level (mmol/L)	2.3 [1.2–4]	2.7 [1.5–4]	2.3 [1.2–6.5]	1.7 [1.2–3.2]	0.315
Platelet count (G/L)	204 [97–307]	170 [84–218]	204 [90–340]	230 [141–325]	0.113
Hematocrit level (%)	29.3 [26–31.6]	30.3 [26.8–32.7]	29.7 [27.3–31.6]	27.7 [25.3–31.1]	0.284
Leucocyte count (G/L)	13.9 [8.9–21]	14.1 [7.1–17.7]	15.6 [8.9–23.2]	13 [9.2–20.3]	0.373
Glasgow Coma Scale score	15 [13–15]	15 [13–15]	15 [13–15]	15 [13–15]	0.661
Bilirubin level (mg/dL)	12 [8–20]	12.5 [9–20]	12 [9–29]	12 [7–19]	0.657
Prothrombin time (%)	66 [52–78]	66 [57–78]	64 [47–81]	67 [57–78]	0.606
Proteinemia (g/L)	56 [47–63]	46.5 [40.5–60.5]	55.5 [49–64]	59 [51–64.5]	0.007

Results are expressed as the median [25th–75th percentiles] or *n* (%) as appropriate

*APACHE* Acute Physiology and Chronic Health Evaluation, *C*_max_ peak plasma concentration, *PaO*_*2*_ */FiO*_*2*_ partial pressure of oxygen in arterial blood/fraction of inspired oxygen

**Table 2:** Baseline patient characteristics at intensive care unit admission and at inclusion (survivors/non-survivors)

Characteristics	Survivors (*n* = 64)	Non-survivors (*n* = 46)	*P*
At ICU admission			
Age (years old)	60 [45–69]	63 [57–73]	0.03
Male sex	49 (76.6)	29 (63)	0.12
Weight (kg)	70 [60–80]	70 [58–79]	0.84
Body mass index (kg/m^2^)	24.6 [20.7–27.8]	24.4 [20.7–28]	0.58
APACHE II	21 [17–25]	26 [22–29]	0.0007
Simplified Acute Physiology Score 2	52 [41–60]	57 [45–73]	0.05
History of congestive heart failure	6 (9.4)	6 (13)	0.54
Liver cirrhosis	0	4 (8.7)	0.03
Chronic obstructive pulmonary disease	15 (23.4)	11 (23.9)	0.96
Chronic kidney disease requiring dialysis	4 (6.3)	5 (10.9)	0.38
Cancer (<3 months)	11 (17.2)	5 (10.9)	0.35
Diabetes mellitus	15 (23.4)	24 (52.2)	0.002
Immunodepression	16 (25)	8 (17.4)	0.34
At the day of aminoglycoside administration			
Day since admission	1 [0–5]	2 [0–7]	0.32
Emergent surgery	17 (26.6)	8 (17.4)	0.26
Sequential Organ Failure Assessment score	9 [7–10]	10 [9–13]	0.004
Weight (kg)	70 [60–81]	70.5 [60–80]	0.92
Weight gain since ICU admission (kg)	0 [0–2]	0 [0–3]	0.62
Extracorporeal membrane oxygenation	7 (10.9)	8 (17.4)	0.33
Catecholamines	49 (76.6)	40 (87)	0.17
Norepinephrine (µg/kg/min)	0.26 [0.04–0.61]	0.83 [0.41–1.65]	0.0002
PaO_2_/FiO_2_ ratio	200 [140–280]	147 [103–259]	0.06
Renal replacement therapy	19 (29.7)	21 (45.7)	0.09
Glomerular filtration rate (mL/min)	53.5 [29.7–90.4]	29.8 [0–65.9]	0.008
Lactate level (mmol/L)	1.7 [1.2–3.1]	3.2 [1.4–7]	0.01
Platelet count (G/L)	205 [105–339]	201 [86–270]	0.47
Hematocrit level (%)	29.2 [26–31.3]	29.7 [26–32.4]	0.54
Leucocyte count (G/L)	15.6 [9.4–22.3]	13.4 [7.8–18.7]	0.22
Glasgow Coma Scale score	15 [14, 15]	15 [12–15]	0.23
Bilirubin level (mg/dL)	12 [8–17]	145 [9–26]	0.22
Prothrombin time (%)	72 [57–81]	60 [47–75]	0.01
Proteinemia (g/L)	58 [49–64]	52 [46–62]	0.1
Amikacin *C*_max_ between 60 and 80 mg/L	34 (53.1)	13 (28.3)	0.009

Results are expressed as the median [25th–75th percentiles] or *n* (%) as appropriate

*APACHE* Acute Physiology and Chronic Health Evaluation, *C*_max_ peak plasma concentration, *PaO*_*2*_ */FiO*_*2*_ partial pressure of oxygen in arterial blood/fraction of inspired oxygen

**Table 3:** Sites of infection and isolated micro-organisms

	*N*
Sites of infection	110
Pulmonary	57
Catheter	9
Intra-abdominal	13
Skin and soft tissue	6
Urinary tract	6
Other	3
Unknown	16
Bacteraemia	32
Isolated micro-organisms	108
Cocci	24
*Staphylococcus aureus*	13
Other staphylococci	3
*Enterococcus* spp.	5
*Streptococcus* spp.	3
Bacilli	84
Enterobacteriaceae	56
*Escherichia coli*	15
*Enterobacter* spp.	18
*Serratia marcescens*	3
*Klebsiella* spp.	14
Other Enterobacteriaceae	6
*Pseudomonas aeruginosa*	21
*Acinetobacter baumannii*	2
Other bacilli	5
None	36

The median age was 61 [51–70], the median Simplified Acute Physiology Score 2 at admission was 54 [41–68], and the median SOFA score on the day of amikacin administration was 10 [8–11]. The sources of infection were respiratory in 57 cases (51.8%), intra-abdominal in 16 cases (14.5%) and catheter related in 9 cases (8.2%). Bacteraemia was present in 32 cases (29.1%) (Table 3).

The most frequently isolated bacteria were *P. aeruginosa* (19.1%), *Enterobacter* spp. (16.7%) and *Escherichia coli* (13.9%) (Table 3).

### Amikacin dosages

The median dose of amikacin was 30 [29.2–30.6] mg/kg. Pharmacodynamic amikacin targets (60 mg/L < *C*_max_ < 80 mg/L) were achieved in 46 of the 110 patients (41.8%). Forty-four of the 110 patients (40%) had a *C*_max_ > 80 mg/L, and 20 of the 110 patients (18.2%) had a *C*_max_ < 60 mg/L. No patient had a *C*_max_ < 30 mg/L (Table 4).

**Table 4:** Amikacin pharmacokinetic/pharmacodynamic parameters

Variable	*n* = 110
Dose (mg)	2100 [1800–2400]
Dose (mg/kg)	30 [29.2–30.6]
Peak plasma concentration (mg/L)	75 [66.1–86.1]
Patients with a peak concentration >80 mg/L	44 (40)
Patients with a peak concentration between 60 and 80 mg/L	46 (41.8)
Patients with a peak concentration <60 mg/L	20 (18.2)
Patients with a peak concentration <30 mg/L	0
Trough concentration >2.5 mg/L^a^	51 (78.5)

Results are expressed as the median [25th–75th percentiles] or *n* (%)

^a^Trough concentration was not measured for 45 patients

Trough concentration was measured for 65 patients (68.2%). Among these, 51 (78.5%) had a trough concentration >2.5 mg/L.

### Prognosis

The in-ICU mortality rate was 41.8%. A univariate analysis revealed that the amikacin *C*_max_ was not associated with a reduction in the in-ICU mortality rate (*C*_max_ was 73.6 [65.9–83.6] mg/L in survivors and 81.9 [66.5–89.7] mg/L in non-survivors, *P* = 0.12).

The univariate analysis also showed that the in-ICU mortality rate was lower in the group of patients with a *C*_max_ between 60 and 80 mg/L (28.3%) than in patients with a *C*_max_ > 80 mg/L (56.8%, *P* = 0.006).

Moreover, there was no statistically significant difference of mortality between the group of patients with a *C*_max_ between 60 and 80 mg/L and the group of patients with a *C*_max_ < 60 mg/L (40%, *P* = 0.18).

The univariate analysis of trough concentration measurements in 75 of the 110 patients (68.2%) revealed that a higher amikacin trough concentration was not associated with a higher in-ICU mortality rate (5.2 [2.3–10.4] mg/L in survivors versus 8.6 [4.2–14.3] mg/L in non-survivors, *P* = 0.058).

The proportion of micro-organisms with a greater than usual amikacin MIC (i.e. non-fermenting Gram-negative bacilli) in non-survivors was similar to that in survivors (16 of 64 in survivors group versus 6 out of 46 in non-survivors group, *P* = 0.12).

The other risk factors found to be predictive of in-ICU mortality (based on a bivariate analysis with *P* < 0.1) are exposed in Table 2.

Following multivariate analysis, compared to a *C*_max_ between 60 and 80 mg/L, *C*_max_ > 80 mg/L was associated with a higher probability of mortality (OR 95% CI 3.96 [1.54–10.2], *P* = 0.004), and *C*_max_ < 60 mg/L was not associated with a higher probability of mortality (OR 95% CI 1.92 [0.46–8.24], *P* = 0.4). The multivariate analysis also revealed that the factors independently associated with a higher mortality rate were a higher age (*P* = 0.02) and a higher norepinephrine dose (*P* = 0.0001) (Table 5).

**Table 5:** Multivariate analysis of risk factors for in-intensive care unit mortality

Variables	Adjusted odds ratio (CI 95%)	*P* value
Age (per year increment)	1.044 (1.01–1.08)	0.02
Norepinephrine (per µg/kg/min increment)	3.94 (1.9–8.15)	0.0001
Amikacin *C*_max_		
Between 60 and 80 mg/L	Reference	Reference
<60 mg/L	1.92 (0.46–8.24)	0.4
>80 mg/L	3.96 (1.54–10.2)	0.004
Prothrombin time	0.98 (0.96–1.01)	0.118
PaO_2_/FiO_2_ ratio	0.99 (0.99–1)	0.28
Diabetes mellitus	1.6 (0.59–4.38)	0.32
Glomerular filtration rate (mL/min)	0.99 (0.98–1.01)	0.43
Lactate level	1.01 (0.89–1.14)	0.53
SOFA	1.03 (0.81–1.3)	0.18

The Hosmer–Lemeshow goodness-of-fit test *P* value was 0.248. The Nagelkerke and Cox/Snell *R* ^2^ were, respectively, 0.347 and 0.258

*CI* confidence intervals, *C*_max_ peak plasma concentration, *PaO*_*2*_ */FiO*_*2*_ partial O_2_ pressure in arterial blood/fraction of the inspired oxygen ratio, *SOFA* Sequential Organ Failure Assessment

In survivors, acute kidney injury was not associated with a *C*_max_ > 80 mg/L (15.8% of patients who developed acute kidney injury had a *C*_max_ > 80 mg/L, while 14.2% who did not had a *C*_max_ > 80 mg/L, *P* = 0.88).

An amikacin trough concentration >2.5 mg/L was not significantly associated with acute kidney injury (*P* = 0.26).

## Discussion

To our knowledge, this is the largest study evaluating the incidence and impact on in-ICU mortality of an amikacin *C*_max_ achieved with a high loading dose of 30 mg/kg. Roger et al. and Galvez et al. evaluated the effects of a dose of 30 mg/kg of amikacin in 47 and 33 patients, respectively, but did not assess the impact of amikacin concentration on mortality [8, 9]. In these studies, the median *C*_max_ obtained with a loading dose of 30 mg/kg of amikacin was 75 mg/L. This is consistent with previous studies that reported values ranging from 72.1 to 75.8 mg/L [8, 9]. We found that, with a loading dose of 30 mg/kg of amikacin, the pharmacodynamic *C*_max_ target (i.e. >60 mg/L for the less susceptible bacteria) was frequently achieved (81.2% cases), a rate that is consistent with previous studies [8, 9].

Studies by Taccone et al. [7] and de Montmollin et al. [6], in which amikacin concentration following a loading dose of 25 mg/kg was evaluated, found a high frequency of underdosing (>30%). These results prompted an increase in the loading dose of amikacin with the aim of achieving a *C*_max_ > 60 mg/L in the highest number of patients [8, 9].

In our study, the amikacin *C*_max_ between 60 and 80 mg/L recommended by French guidelines [4] was associated with a reduction in the in-ICU mortality rate. In theory, a *C*_max_ of amikacin between 60 and 80 mg/L would be optimal for treating sepsis caused by less susceptible Enterobacteriaceae and non-fermenting Gram-negative bacterial infections (such as *P. aeruginosa*) with a MIC of 8 mg/L. This is highlighted in a study by Dubois et al. [14], in which 61% of the isolated *P. aeruginosa* strains had a MIC of 8 mg/L. However, in patients with severe sepsis or septic shock, aminoglycosides are frequently used in empiric antimicrobial treatment without the causal micro-organisms and their MICs being identified. One of the major potential advantages of using aminoglycoside combination therapy is to broaden antibiotic treatment in cases of resistant bacteria, which may be found in ICUs [15, 16]. Combination therapy can increase the success of empiric therapy in up to 20% of patients [17, 18]. A high *C*_max_ is therefore likely necessary given that *P. aeruginosa* is one of the most frequently isolated micro-organisms, even in cases of early-onset sepsis [19, 20].

We agree with Roger et al. [8] that a lower amikacin *C*_max_ may be enough to treat sepsis. In their study, all isolated strains had an amikacin MIC ≤ 4 mg/L, meaning that a *C*_max_ between 32 and 64 mg/L would be sufficient to meet pharmacokinetic/pharmacodynamic parameters in all patients (i.e. *C*_max_/MIC > 8) [8]. In the present study, we found that with a high dose of 30 mg/kg of amikacin, *C*_max_ > 80 mg/L was frequently observed and was associated with higher mortality rates. We did not identify a clear hypothesis to explain the relationship between amikacin concentration >80 mg/L and mortality. Similar mortality results were found in a small randomised study of 99 patients by Galvez et al. [9] compared three dosing regimens (15, 25 and 30 mg/kg) of amikacin in patients with severe sepsis or septic shock. In the 15, 25 and 30 mg/kg amikacin-treated groups, the study found that a *C*_max_ > 60 mg/L was reached in 0, 39 and 76% of patients, respectively, but the mortality rate was 0, 9.1 and 22.2%, respectively. This study was not, however, designed to statistically analyse mortality. We found that patients with amikacin overexposures tended to have greater amikacin trough concentrations (*P* = 0.08). It is well established that aminoglycosides are associated with nephrotoxicity [21, 22], and that a high amikacin *C*_max_ in ICU is associated with higher trough-level concentrations [6]. Nevertheless, it is difficult to consider these high *C*_max_ as “overexposure” and further studies are needed. Moreover, recent in vitro and in vivo studies suggest that higher aminoglycosides exposure should be targeted for difficult to treat pathogens [23, 24]. To date, there is a lack of data on aminoglycoside-related toxicity in once-daily dose and short-duration aminoglycoside therapy. The present study was not designed to analyse and evaluate the association between acute kidney injury and trough concentration or aminoglycoside *C*_max_, in that trough concentrations were not measured in all patients, as suggested by French guidelines [4]. Furthermore, previous studies suggest that aminoglycoside-associated acute kidney injury cannot be solely attributed to aminoglycosides, because other factors are frequently associated with acute kidney injury in the ICU, including sepsis, septic shock or nephrotoxic drugs [25, 26].

Despite the current trend to increase doses of antibiotics in ICUs for pharmacokinetic/pharmacodynamic purposes, no beneficial effect on outcome has been clearly demonstrated [6–8]. In clinical practice, in Europe physicians are still using low doses of aminoglycosides, as shown by a recent survey of antimicrobial prescribing practices in ICUs, where aminoglycosides were administered at “low” doses (5 mg/kg for gentamicin and 15 mg/kg for amikacin) [27].

This study has severe limitations. Given that it was not a randomised controlled trial comparing different loading regimens, we cannot conclude a direct causal relationship between the amikacin *C*_max_ and the mortality rate. We did not evaluate the subsequent amikacin *C*_max_ after the first injection of the study, and MIC of isolated bacteria was not determined for amikacin. Although the study population may be considered small, to our knowledge this is the largest cohort to have been selected for evaluating the impact of high doses of 30 mg/kg of amikacin in patients with severe sepsis or septic shock [8, 9]; moreover, other studies confined their analysis to pharmacokinetics/pharmacodynamics parameters [8, 9]. We agree that analysing pharmacokinetics/pharmacodynamics parameters of medications is one of the first steps before launching an impact study [28]. Nevertheless, aminoglycosides are associated with well-known and severe side effects and analysing outcomes when increasing usual doses is indispensable. Previous studies reported that when high doses of aminoglycosides are administered, sampling within 90 min of the infusion provides information that leads to the overestimation of the peak serum concentration/minimum inhibitory concentration ratio and to the inaccurate calculation of pharmacokinetic parameters [29, 30]. Nevertheless, these studies were confined to gentamicin concentrations in a limited number of healthy volunteers (<13) [29, 30]. By contrast, recent studies [6–10] have reported measurements of amikacin *C*_max_ performed 30 min after infusion, as suggested by French guidelines [4].

## Conclusion

This study suggests that with a high loading dose of 30 mg/kg the amikacin *C*_max_ between 60 and 80 mg/L recommended by French guidelines is associated with reduced in-hospital mortality, but that it is difficult to achieve in ICU patients (<50%). Amikacin overexposure (i.e. *C*_max_ > 80 mg/L) was frequent and potentially associated with increased mortality.

## Abbreviations

APACHE: Acute Physiology and Chronic Health Evaluation
*C*_max_: peak plasma concentration
ICU: intensive care unit
EUCAST: European Committee on Antimicrobial Susceptibility Testing
MIC: minimal inhibiting concentration
SAPS: Simplified Acute Physiology Score
SOFA: Sequential Organ Failure Assessment
